# Postoperative Cognitive Impairment and Pain Perception after Abdominal Surgery—Could Immersive Virtual Reality Bring More? A Clinical Approach

**DOI:** 10.3390/medicina59112034

**Published:** 2023-11-17

**Authors:** Gabriela Droc, Sebastian Isac, Elisabeta Nita, Cristina Martac, Miruna Jipa, Diana Irene Mihai, Cristian Cobilinschi, Andrada-Georgiana Badea, Damiana Ojog, Bogdan Pavel, Maria-Daniela Tanasescu, Teodora Isac

**Affiliations:** 1 Department of Anesthesiology and Intensive Care, Fundeni Clinical Institute, Faculty of Medicine, Carol Davila University of Medicine and Pharmacy, 020021 Bucharest, Romania; gabriela.droc@umfcd.ro; 2 Department of Anesthesiology and Intensive Care I, Fundeni Clinical Institute, 022328 Bucharest, Romania; beti_iris@yahoo.com (E.N.); christtina.martac@yahoo.com (C.M.); mirunaa.jipa@gmail.com (M.J.); diana.mihai@stud.umfcd.ro (D.I.M.); damiana.p.ojog@stud.umfcd.ro (D.O.); 3 Department of Physiology, Faculty of Medicine, Carol Davila University of Medicine and Pharmacy, 020021 Bucharest, Romania; 4 Department of Orthopedics and Anesthesiology, Faculty of Medicine, Carol Davila University of Medicine and Pharmacy, 020021 Bucharest, Romania; cristian.cobilinschi@stud.umfcd.ro; 5 Bucharest Clinical Emergency Hospital, 014461 Bucharest, Romania; 6 Department of Medical Semiology, Discipline of Internal Medicine I and Nephrology, Faculty of Medicine, Carol Davila University of Medicine and Pharmacy, 020021 Bucharest, Romania; maria.tanasescu@umfcd.ro; 7 Department of Internal Medicine II, Faculty of Medicine, Carol Davila University of Medicine and Pharmacy, 020021 Bucharest, Romania; teodora.isac@drd.umfcd.ro; 8 Department of Internal Medicine II, Fundeni Clinical Institute, 022328 Bucharest, Romania

**Keywords:** virtual reality, postoperative cognitive dysfunction, postoperative delirium, postoperative pain, general anesthesia

## Abstract

*Background and Objectives*: Impaired cognition and pain after surgery contribute to prolonged hospital stays and increased mortality rates. Thus, the development of preemptive algorithms for reducing their impact should be prioritized. The main objectives of the present study were to evaluate the efficiency of using virtual reality (VR) to treat postoperative cognitive decline and pain perception. *Materials and Methods:* The study was a prospective, monocentric, clinical study that included 51 patients who have undergone major abdominal surgery. The patients were divided into two groups: Control (n = 25) and VR (n = 26). The VR sessions consisted of 5–8 min exposure at 24–48 h after surgery. We considered the outcome variables, the mini-mental state examination, and visual analogue scale at 24–48 h after surgery. The dependent variables were age, social status, educational level, and duration of surgery. *Results*: We did not observe any differences in postoperative cognition deficit with regard to VR. The VR, however, successfully reduced postoperative pain intensity. Moreover, the patients’ age, surgery duration, level of education, and social status influenced the MMSE score at 24–48 h after surgery. *Conclusions*: Even if using VR does not alleviate short-term postoperative cognitive impairments, it could affect pain perception. Further studies are needed to support the use of VR in perioperative contexts.

## 1. Introduction

Postoperative cognitive decline is a complex pathology that usually arises after surgery and includes postoperative delirium (POD) and postoperative cognitive dysfunction (POCD). The first signs of POCD can appear hours after a surgical procedure and even persist for months. POCD is characterized as a decline in cognitive function, and the manifestation range includes impaired memory, speech, and reasoning, as well as difficulty concentrating, and POCD has associated with a prolonged time before returning to work, early retirement, and a rise in mortality rates [1]. The diagnosis of POCD is mainly based on subjective neuropsychological testing tools [2].

Postoperative delirium (POD) is an acute and severe condition that usually appears at days 1–3 after a procedure. This complication is characterized by fluctuating levels of consciousness and impairment in thinking and attention, and it is also associated with high mortality and morbidity (persistent brain dysfunction such as dementia, extensive hospitalization, or a higher risk of institutionalization). Even though it has been shown that POD and POCD are interrelated, the former is considered an important risk factor for the latter in the first postoperative month [3]. Data on this, however, are still lacking.

Because early physical and psychological recovery after surgery is, nowadays, much sought, specialists should prioritize new strategies to discover the most suitable preemptive method for POD and POCD. Since the diagnosis approaches are debatable and heterogeneously applied, further preemptive non-pharmacological therapeutic strategies have become very popular worldwide [4]. Clinical psychologists play a critical role in various non-pharmacological approaches against postoperative cognitive decline [5]. The main non-pharmacological strategies involve the following: approaches regarding sleep hygiene, maintaining social contact, occupational therapy, suitable postoperative pain management [6]. However, these approaches have not yet been able to reduce the incidence of patients experiencing cognitive decline after undergoing major surgical procedures. Thus, the use of virtual reality (VR) in this context, could provide satisfactory results, even if its use it still not standard in the medical field [7]. The main two forms of VR suitable in ICU settings are immersive or non-immersive VR [7]. Immersive VR involves the use of a head-mounted display (i.e., VR headset or glasses); the main difference between immersive and non-immersive VR is that the former provides a more realistic experience due to the 360° images on display. However, thus far, no clinical trials have proved the efficacy of using any form of VR technology in managing postoperative cognitive decline.

The mechanisms underlying the pathology of postoperative cognitive decline are multifactorial, with the main culprit being anesthesia-induced neuromodulation. General anesthesia leads to a rise in the permeability of mitochondrial membranes, which subsequently malfunction and disrupt the balance of calcium homeostasis in nerve cells and also impair energy-related functions. Neuroinflammation in the context of inflammatory response due to surgical stress could influence the magnitude of postoperative cognitive decline [8]. Inflammation leads to a massive release of pro-inflammatory cytokines like Interleukin-6 and Tumor Necrosis Factor-alpha into the bloodstream, which disrupt the blood–brain barrier. Thus, macrophages and activated leukocytes’ migration leads to the activation of microglia and astrocytes [8]. Moreover, surgical stress stimulates the sympathetic nervous system, the hypothalamic–pituitary–adrenal (HPA) axis and apoptosis-related proteins synthesis. Blocking the stellate ganglion could prevent POCD by reducing the surgical stress [9].

The cholinergic system and the dopaminergic, adrenergic and GABAergic ones have a significant role in maintaining appropriate cognitive function. The imbalance of the neurotransmitters can contribute to the pathogenesis of POCD; the accumulation of dopamine increases excitability, and inadequate interaction between dopamine and acetylcholine leads to excitation–inhibition dissociation [10]. The expression levels of agonists of the central cholinergic system receptors may increase, while those of antagonists may decrease, deteriorating proper memory function, learning, or other cognitive tasks. Moreover, there is an interaction between β-amyloid peptide and nicotinic receptors, leading to their down-regulation [10]. Furthermore, inappropriate pain management could lead to cognitive impairments in perioperative settings. Pain leads to further stress and the activation of the adrenergic system. Studies have shown that by blocking the sympathetic system’s overactivation, postoperative cognition can be significantly improved [9]. Intraoperative awakening could also negatively contribute to the patient’s postoperative condition [1]. Pain plays an important role in the development of POCD. High levels of pain lead to increased anxiety, which in turn could aggravate the postoperative cognitive dysfunction of the patient [11]. Untreated, acute postoperative pain which lasts longer than 3 months becomes chronic, which is also an important risk factor for postoperative cognitive impairments. Moreover, untreated preoperative chronic pain leads to a higher incidence of POCD after undergoing general anesthesia for major surgical procedures [12].

The use of VR for ameliorating pain perception is still controversial. VR changes pain perception via multiple mechanisms, like distraction and pain control, although some studies also suggest that it could influence the neurophysiology of pain [13,14]. Nociceptive stimulus is conducted towards the cortex by the ascending pain pathways, and the pain sensation can be modulated by the descending pain pathways emerging from the periaqueductal gray matter (PAG). While senses like emotion, concentration, and memory might interfere with pain sensation by modifying the signals in ascending pain pathways, Gold et al. suggest that the regions of the cortex responsible for emotion and concentration also activate PAG in order to reduce pain intensity [15]. Further research is needed to confirm whether VR can activate these nervous impulses. Nevertheless, reducing pain by any means leads to an improvement in the patient’s postoperative mental state.

Even if the pathophysiology of these conditions is not fully understood, a number of risk factors have been implicated, such as age, education, systemic inflammation, duration of the surgery, time under anesthesia, blood oxygen saturation during brain surgery, hypertension, atherosclerosis, liver cirrhosis, and preexisting neurological deficits [3,16,17].

The aim of this study was to assess the impact of immersive virtual reality (VR) on short-term postoperative cognitive decline and pain intensity. The secondary objective was to identify the other possible risk factors for postoperative cognitive decline.

## 2. Materials and Methods

### 2.1. Study Design: Inclusion/Exclusion Criteria

This study was a monocentric, prospective, randomized clinical study including patients who have undergone major abdominal surgery. The study was conducted in accordance with the Declaration of Helsinki and approved by the Local Ethics Committee (66776/27.10.2021) Informed consent was obtained from all participants, and the possibility of being exposed to immersive VR was acknowledged. We considered major abdominal surgery, as well as the surgical procedures for oncologic and/or non-oncologic pathologies performed via a classical approach (non-laparoscopic), like hepatobiliary, pancreatic, and/or digestive tract surgery. A total of 51 patients were enrolled in our study from August 2022 to April 2023. The included patients were over 50 years old and exposed to general surgery procedures that lasted over 3 h. The exclusion criteria were as follows: patients’ refusal; minor surgical procedures like cholecystectomy, appendicectomy, or inguinal hernia repairs; and postoperative complications that required prolonged intubation.

Through using a simple randomization technique, the patients were randomly sorted into two groups: Control (n = 25) and VR (n = 26) groups. The outcome variables were Mini Mental State Examination (MMSE) score and VAS (Visual Analog Scale) score at 24 and 48 h, respectively, as used in [18].

Additionally, we considered the following qualitative and quantitative parameters as independent variables: age, duration of surgery, educational level, social status. For educational level, we considered the last school the patients’ graduated from and divided them into the following groups: primary (n = 10), secondary (n = 24), and higher education (n = 17). For social status, we divided the patients into two groups: alone (n = 17) or living with their family (n = 34). The general anesthetic was administered at the discretion of the anesthesiologist, and the degree of intraoperative analgesia was titrated with NOL Monitoring^®^ (Nociception Level) (Medtronic plc, Dublin, Irland) as previously described [16]. The patients received a balanced general anesthetic. For anesthesia induction, we used propofol (2 mg/kg) (Fresenius Kabi GmbH, Graz, Austria) as a hypnotic, fentanyl (2–3 µg/kg) (Chiesi Pharmaceuticals GmbH, Vienna, Austria) as an analgetic, and rocuronium (0.6 mg/kg) (N.V. Organon, Oss, The Netherlands) as a muscle relaxant. Furthermore, the general anesthesia was maintained using sevoflurane (1.9–2.3% in expired gas) (S.C. Rompharm Company S.R.L, Otopeni, Romania) as a hypnotic, while for analgesia and muscle relaxation, the patients received fentanyl and rocuronium, titrated to effect, respectively. Additionally, some patients received a neuraxial block, depending the anesthesiologist’s judgment and surgical procedure. For the neuraxial block, ropivacaine 0.5% (S.C. Fresenius Kabi Romania S.R.L, Brasov, Romania) (bolus technique, titrated to effect) was used intraoperatively. The postoperative analgesia regimen was standardized in accordance with the local guidelines [18]. The patients received acetaminophen (3 g/day) (S.C. Santa S.A., Brasov, Romania) and metamizole (4 g/day) (S.C. Zentiva S.A., Bucharest, Romania). As a rescue therapy, the patients received morphine (0.1 mg/kg) (Lannacher Heilmittel G.m.b.H, Lannach, Austria), titrated to effect.

The MMSE score was assessed by a certificated clinical phycologist, while VAS score was documented by the ICU nurse at 24–48 h after surgery. The patients in the VR group were exposed to 5 to 8 min immersive VR sessions carried out in accordance with the methods outlined in [19]. The immersive VR consisted of exposure to nature landscapes like hills, forests, and seas and/or plain images in accordance with the patients’ wishes. The VR settings were established by the psychologist and based on the specific social background of each patient.

### 2.2. Statistical Analysis

For our statistical analysis and graphical representations, we used GraphPad 9.0 (GraphPad Software Inc., San Diego, CA, USA). We evaluated the groups for the normal distribution using the D’Agostino–Pearson omnibus normality test and the Shapiro–Wilk test. For our comparative analysis, we considered the mean values of the MMSE and VAS scores at 24 and 48 h after surgery with regard to the VR use, social status, and the last school they graduated from. The MMSE score retained values between 0 and 30, while the VAS score ranged between 1 and 10. The results were expressed as means ± SEM. We considered a two-sided *p* value below 0.05 to be statistically significant.

For our correlative analysis, we considered the age (years) and the surgery duration (hours) as independent variables and MMSE score at 24 and 48 h as outcome variables. Pearson correlation coefficients, r, and R squared values, R^, were determined. We assumed a R^ value above 0.15 to be clinically significant by a two-sided *p* value under 0.05.

## 3. Results

### 3.1. The Impact of VR on the MMSE and VAS Scores

**Figure 1:**
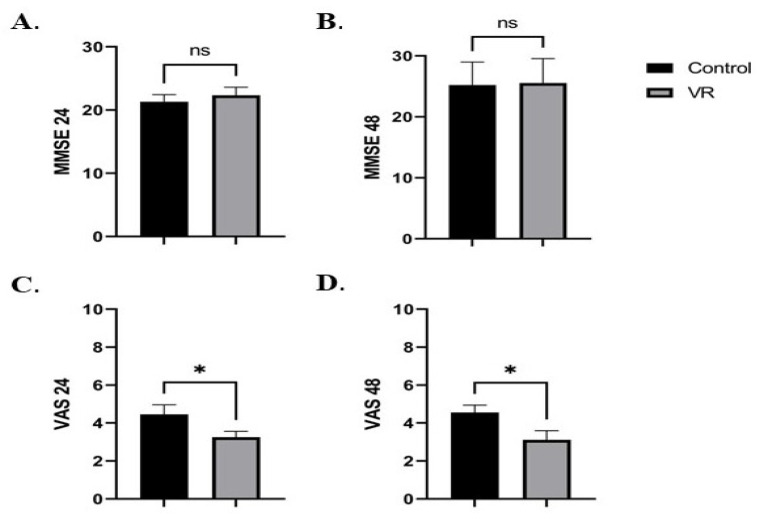
The impact of VR on MMSE (**A**,**B**) and VAS scores (**C**,**D**) at 24 and 48 h after surgery. The results are expressed as mean ±SEM. * represents a *p* value between 0.05 and 0.01, and ns means non-significant.

The results regarding the MMSE scores at 24 and 48 h with regard to VR are revealed in Figure 1A,B. We did not observe any differences between the VR and Control groups either 24 or 48 h after surgery: 22.3 ± 6.3 vs. 21.3 ± 1.2 (*p* = ns) and 25.5 ± 0.9 vs. 25.2 ± 0.8 (*p* = ns). The mean ±SEM VAS score was statistically significantly lower in the VR group compared to the Control group at 24 and 48 h after surgery: 3.2 ± 0.3 vs. 4.4 ± 0.5 (*p* = 0.04) and 3.1 ± 0.4 vs. 4.5 ± 0.3 (*p* = 0.04) (Figure 1C,D).

### 3.2. Other Risk Factors for POCD

**Figure 2:**
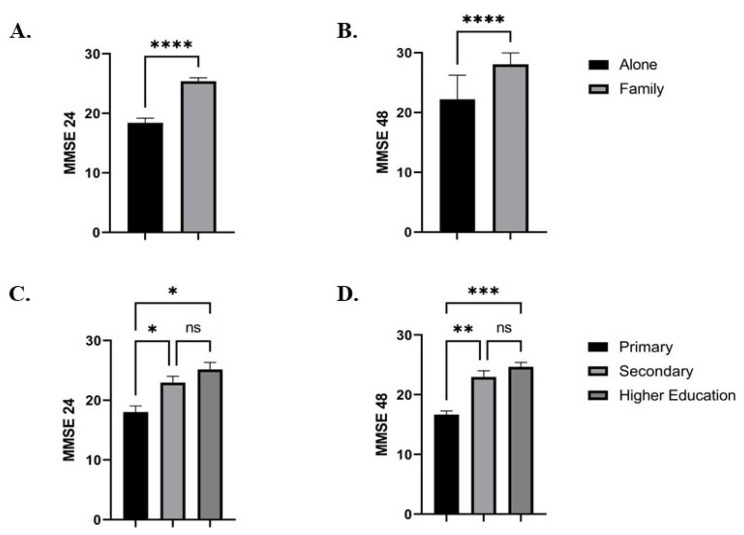
The MMSE scores at 24 and 48 h after surgery with regard to social status (**A**,**B**) and educational level (**C**,**D**). The results are expressed as mean ± SEM. * represents a *p* value between 0.05 and 0.01, **—*p* value between 0.01 and 0.001, ***—*p* value between 0.001 and 0.0001, ****—*p* value ˂ 0.0001, and ns means non-significant.

Figure 2 represents the impact of the social status (A,B) and educational level (C,D) on the MMSE score at 24 and 48 h, respectively. We observed an increase in MMSE score at 24 and 48 h, respectively, in patients living with their families compared to the patients living alone: 25.3 ± 0.5 vs. 18.4 ± 0.7 (*p* ˂ 0.0001) and 28.0 ± 0.4 vs. 22.2 ± 1.0 (*p* ˂ 0.0001) (Figure 2A,B).

Regarding the patients’ educational levels, we found a reduction in MMSE score at 24 h after surgery when comparing the primary education group with the secondary and higher education groups, respectively: 18.0 ± 1.0 vs. 22.9 ± 1.0 (*p* = 0.03) and 18.0 ± 1.0 vs. 25.1 ± 1.1 (*p* = 0.01). No differences were observed between the secondary and higher education groups in MMSE score at 24 h after surgery: 22.9 ± 1.0 vs. 25.1 ± 1.1 (*p* = ns) (Figure 2C). Similar results were observed among the groups at 48 h. The MMSE score at 48 h after surgery was reduced in the primary education group compared to both the secondary and higher education groups, respectively: 16.6 ± 0.6 vs. 22.9 ± 1.0 (*p* = 0.004) and 16.6 ± 0.6 vs. 24.6 ± 0.7 (*p* = 0.009). No differences were observed in MMSE score between the secondary and higher education groups: 22.9 ± 1.0 vs. 24.6 ± 0.7 (*p* = ns) (Figure 2D).

**Figure 3:**
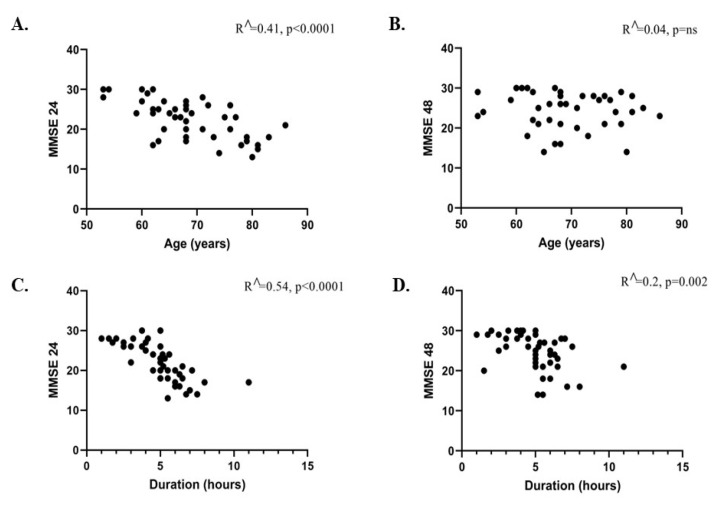
The correlation between patient age (**A**,**B**) and surgery duration (**C**,**D**) and MMSE score at 24–48 h after surgery. R^ represents the correlation coefficient.

Additionally, we observed that the patients age and duration of surgery modified the MMSE score 24–48 h after surgery (Figure 3A–D). Patient age was negatively correlated with MMSE score at 24 h after surgery (R^ = 0.41, *p* ˂ 0.0001) but not at 48 h after surgery (R^ = 0.04, *p* = ns) (Figure 3A,B). Moreover, surgery duration was also negatively correlated with MMSE score at 24 h (R^ = 0.54, *p* ˂ 0.0001) and 48 h (R^ = 0.02, *p* = 0.002) after surgery (Figure 3C,D).

## 4. Discussion

The majority of surgical procedures are associated with complications such as acute postoperative pain or cognitive impairment. Clinicians have implemented new techniques to avoid these by using novel technologies such as VR.

The more that clinical trials use VR to reduce acute postoperative pain, the clearer it becomes that this method does have clinically significant results [20,21]. The standard therapy for immediate postoperative pain involves the use of opioids. By conducting a randomized clinical trial, Payne et al. came to the conclusion that not only did immersive VR reduce the pain experienced by women undergoing gynecological laparoscopic surgery but also the dose of opioids received after surgery was also significantly lower [20]. The same results were found by Olbrecht et al., reinforcing to the fact that pain is mostly diminished by distraction and redirection [22]. Many studies support the theory that distraction leads to most of the analgesia provided by VR [23]. Whether it works directly or indirectly on signaling pathways of pain, attention, or emotion, it is clear that the affective component of pain is influenced by distraction via VR use [23,24]. Another way of influencing painful sensations with VR is pain control, wherein the subject is invited to control the pain while immersed in the virtual reality. The results related to this show that through guided-relaxation VR, levels of pain can be significantly lowered, as can be achieved through using distraction VR. Anxiety levels can also be decreased though VR use [23]. In terms of incorporating VR more into the treatment of pain outside the acute setting, Gupta et al. proposed that VR technology could also induce neurophysiologic changes in order to diminish the perception of pain [14]. Their results showed significantly higher pain thresholds for patients who attempted to control their pain via VR versus those who did not use VR, but no statistically significant difference between distraction and pain control was found.

One field in which clinicians have actively involved VR is burn wound care, mostly because, usually, the pain associated with these wounds is resistant to opioid analgesia. By combining VR with pharmacological analgesia, studies have shown that pain sensation is significantly lower in patients undergoing burn wound care than in those who have only received standard analgesia [13,14,22].

Our results are similar to those reported in the literature, showing that VR can significantly influence pain perception. In our study, patients’ pain intensity at 24 and 48 h after surgery (measured using VAS) was statistically lower for those who received VR therapy.

Another affective component which virtual reality seems to influence is anxiety related to pain. Some studies suggest that VR can be successfully used in treating anxiety and phobias [25]. On the other hand, in some cases, levels of anxiety during VR use are higher due to the fact that patients are unable to see the procedure they are undergoing because of the VR glasses [26]. It is well known that the presence of anxiety before and after a medical intervention can also influence the mental state of the patient. Reducing patients’ anxiety before and after surgery might lead to quicker recovery times [10].

Patients’ cognition is also altered by undergoing surgery. There is evidence that VR use could improve hospitalized patients’ mental states. Binbin Zhu et al. recently developed a study protocol in order to research whether VR therapy influences the development of POCD at 1 year after surgery [27].

Nonoperative patients’ mental states can also be altered. In fact, the longer the hospital stay is, the greater the cognitive impairment. Researchers have encouraged using VR for improving the quality of life and mental states of patients who have been (or are set to be) hospitalized for a prolonged period of time [28,29].

While VR is regarded as a support tool for neurological and psychological function in chronic patients out of the operative setting, its potential role in postoperative care requires more research [30,31,32].

Our study on patients undergoing major abdominal surgery did not reveal any changes in the cognitive status of the patients. This could be explained by the short follow-up timeframe (24–48 h). Further clinical studies are needed to verify this study’s findings.

There are other factors that influence the patients’ cognition, whether they benefit from VR or not. Similar to our findings, the literature shows that patients who are younger and have higher educational levels are likely to achieve a faster postoperative recovery [33,34,35]. Educational level could have a higher influence on MMSE scores than age [33]. It has also been noted that elderly patients could find VR settings too complicated, and in such cases, VR use does not appear to provide any improvements to their mental state [32]. Living alone has been shown to be a risk factor for POCD [34]. In our study, there was a statistically significant difference in the MMSE scores in favor of patients living with their families.

### Study Limitations

As no patient with a known neurological pathology was enrolled in this study, we did not perform any cognitive assessment before surgery. Moreover, the short follow-up period could have excluded the possible long-term beneficial effects of VR use on the patients’ cognitive status. Finally, we did not consider the anxiety levels of patients before and after using immersive VR, as this may have led to bias in the results for cognition and pain perception. We do, however, intend to address these issues in future studies.

## 5. Conclusions

Even though postoperative cognitive decline and pain syndrome after major abdominal surgery are difficult to predict, the use of VR could play an important role in reducing their impact, especially in afflicted patients.

## Data Availability

Data are contained within the article.
